# Screening for infectious maternal morbidity - knowledge, attitudes and perceptions among healthcare providers and managers in Malawi: a qualitative study

**DOI:** 10.1186/s12884-022-04583-5

**Published:** 2022-04-26

**Authors:** Emilia Slezak, Holger Unger, Luis Gadama, Mary McCauley

**Affiliations:** 1 Centre for Maternal and Newborn Health, Liverpool School of Tropical Medicine, Liverpool, UK; 2 Department of Obstetrics and Gynaecology, Royal Darwin Hospital, Darwin, Australia; 3 Menzies School of Health Research, Charles Darwin University, Darwin, Australia; 4 Queen Elizabeth Hospital, Blantyre, Malawi; 5 Liverpool Women’s NHS Foundation Trust, Liverpool Women’s Hospital, Crown Street, L8 7SS Liverpool, UK

**Keywords:** Maternal morbidity, Infections, Early warning scores, Healthcare providers, Antenatal care, Postnatal care, SARS-CoV-2

## Abstract

**Background:**

Maternal morbidity and mortality related to infection is an international public health concern, but detection and assessment is often difficult as part of routine maternity care in many low- and middle-income countries due to lack of easily accessible diagnostics. Front-line healthcare providers are key for the early identification and management of the unwell woman who may have infection. We sought to investigate the knowledge, attitudes, and perceptions of the use of screening tools to detect infectious maternal morbidity during and after pregnancy as part of routine antenatal and postnatal care. Enabling factors, barriers, and potential management options for the use of early warning scores were explored.

**Methods:**

Key informant interviews (*n* = 10) and two focus group discussions (*n* = 14) were conducted with healthcare providers and managers (total = 24) working in one large tertiary public hospital in Blantyre, Malawi. Transcribed interviews were coded by topic and then grouped into categories. Thematic framework analysis was undertaken to identify emerging themes.

**Results:**

Most healthcare providers are aware of the importance of the early detection of infection and would seek to better identify women with infection if resources were available to do so. In current practice, an early warning score was used in the high dependency unit only. Routine screening was not in place in the antenatal or postnatal departments. Barriers to implementing routine screening included lack of trained staff and time, lack of thermometers, and difficulties with the interpretation of the early warning scores. A locally adapted early warning screening tool was considered an enabler to implementing routine screening for infectious morbidity. Local ownership and clinical leadership were considered essential for successful and sustainable implementation for clinical change.

**Conclusions:**

Although healthcare providers considered infection during and after pregnancy and childbirth a danger sign and significant morbidity, standardised screening for infectious maternal morbidity was not part of routine antenatal or postnatal care. The establishment of such a service requires the availability of free and easy to access rapid diagnostic testing, training in interpretation of results, as well as affordable targeted treatment. The implementation of early warning scores and processes developed in high-income countries need careful consideration and validation when applied to women accessing care in low resource settings.

**Supplementary Information:**

The online version contains supplementary material available at 10.1186/s12884-022-04583-5.

## Background

Infectious maternal mortality and morbidity is an international public health concern [1–3]. Global estimates suggest that direct (obstetric) infections are the third most common cause of maternal mortality, representing 10·7% of maternal deaths, with the largest toll estimated in low- and middle-income countries (LMICs) at 10·7% compared with high-income countries at 4·7% [1, 3]. Many women experience different severities of infective health disorders during and after pregnancy in LMIC settings and not uncommonly these remain undetected or are recognised late [4–6]. The Global Burden of Disease study estimated that 11·9 million cases of maternal infections occurred in 2017, but data for infectious maternal morbidity in LMICs is lacking [7]. The Sustainable Development Goal 3 is to improve the health and well-being for all at all ages by 2030, and the Global Strategy for Women’s, Children’s and Adolescent’s Health emphasises that all women have the right to, and should obtain, the highest attainable standard of health, including comprehensive and holistic antenatal and postnatal care [8, 9]. Infectious maternal morbidity is associated with adverse consequences for the mother and the baby, both in the short and long term [10] and the prevention, early diagnosis, and prompt management of infection are key factors for reducing related morbidity and mortality during routine maternity care [3].

In LMIC settings interventions to detect, prevent and manage infectious maternal morbidity have largely been focused on malaria, HIV, tuberculosis, syphilis, and more recently the priority intervention has been to prevent and manage the SARS-CoV-2 disease caused by the Covid-19 virus [11, 12]. General maternal infections (such as urinary tract infection, chorioamnionitis, endometritis, mastitis) can lead to sepsis with significant maternal morbidity and adverse clinical outcomes if undetected and untreated [2, 3]; but these infections are less frequently assessed for during routine antenatal or postnatal care in women living in LMICs [4–6]. Many high-income countries have implemented routine screening for maternal infection as part of routine antenatal care by trained healthcare providers including routine screening for HIV, syphilis, hepatitis B, rubella, and asymptomatic urinary tract infection [13–15]. Other examples of screening for infections include the use of early warning screening tools that incorporate clinical indicators such as heart rate, respiratory rate, and temperature in addition to laboratory tests such as white blood cell count, lactate, or C-reactive protein levels to identify women with infection [16–24]. Approaches to screening are often used at inpatient healthcare facility level to detect and help prevent the progression of a mild infection to a more severe infection and can additionally be used to identify the unwell woman with sepsis promptly [16–24]. Early warning screening scores have been shown to reduce infection related morbidity and mortality in high-income and in some LMIC settings [25–30]. There are many international policies and guidelines that promote screening for infections as part of comprehensive routine antenatal and postnatal care [29–31]. However, implementation in LMIC is challenging [32]. Globally, 85% of women attend for antenatal care at least once [33]. It is essential that this does not become a lost opportunity and that healthcare providers are enabled to provide good quality care including a comprehensive assessment and management of infective maternal morbidity during and after pregnancy [4–6, 34]. This has been highlighted recently with the event of the global SARS-CoV-2 pandemic, a likely significant contributor to maternal morbidity and mortality in LMIC settings [35].

This study sought to investigate the knowledge, attitudes, and perceptions of maternity care providers regarding routine screening to detect infectious morbidity during and after pregnancy in Blantyre, Malawi. Enabling factors and barriers to the implementation of infection screening using early warning scores during routine antenatal and postnatal care, including outpatient and inpatient settings, were explored.

## Methods

### Study design and setting

Key informant interviews and focus group discussions were conducted with healthcare providers working in the obstetric department the Queen Elizabeth Hospital, in Blantyre, Malawi, in June 2019 (prior to Covid-19 pandemic caused by SARS-Co-V). This hospital is the largest teaching hospital in the south of Malawi providing routine and specialised antenatal, intrapartum, and postnatal care, in addition to receiving high risk referrals from surrounding healthcare facilities. There are on average 1000 deliveries per month, with a Caesarean rate of 30%. All interviews were held a quiet office in the healthcare facility away from the clinic rooms to ensure privacy.

### Participants

Healthcare providers (doctors, nurses, midwives) were included if they provided routine maternity care (including antenatal and postnatal care) at the chosen study site. Healthcare managers (Ward matron, Head of Department, Head of facility) were included to enable the triangulation of the data and broadened the scope of the topic. Snowballing and opportunistic sampling techniques were employed to identify the participants. Participants were chosen purposively, based on their ability to speak English, and were recruited sequentially until data saturation was met.

### Topic guide

A topic guide was developed and piloted at the study site in Malawi. The topic guide was a flexible tool that enabled the interviewer to capture the healthcare providers’ responses as well as acting as a cue to probe further to understand the participants’ perceptions and beliefs (Supplementary File 1). In addition to sociodemographic questions, the topic guide included five main subject areas: (1) overall understanding of screening for infectious morbidity (2) knowledge and perception of early warning scores; (3) experience and views on use of early warning scores; (4) approaches to management of women with infectious morbidity; and (5) suggestions on how to identify or screen women with possible infectious maternal morbidity.

### Data collection

Key informant interviews and focus group discussions were conducted face-to-face in English, lasted on average 30–45 min, were recorded on a digital recording device, and transcribed on completion. Anonymity and confidentiality with regards to data reporting were emphasised to reassure participants’ confidence in providing honest answers. All participants approached agreed to participate in the study and completed the interviews.

### Analysis

The interviews and focus group discussions were transcribed verbatim by the first author (ES). The first author (ES) and a second reviewer (HWU) independently coded all transcripts. The identified codes were grouped into categories and reviewed by three researchers (ES, HU, MMC) to ensure consistency. This enabled the first extraction of data [36]. Key themes were then discussed and checked by all researchers together to reach consensus. We used the Standards for Reporting Qualitative Research guidelines in reporting the analysis [37].

### Ethics

Ethical approval was granted by the Liverpool School of Tropical Medicine, UK (LSTM14.025) and by the University of Malawi College of Medicine Research and Ethics Committee (COMREC 2724). This research was conducted in accordance with the Declaration of Helsinki. Written informed consent was obtained from all participants of the study.

## Results

### Participants’ characteristics

Twenty healthcare providers and four healthcare managers participated in the study (10 doctors and 14 nurse-midwives; 16 females and 8 males). Two Consultant obstetrician-gynaecologists, four nurse-midwives and four healthcare managers (Head of facility, Head of Department, Matrons) provided key informant interviews and six doctors with varied levels of experience (junior doctor, specialist registrar, and consultant) and eight midwives participated in two separate focus group discussions. The age of participants ranged between 25 and 65 years, with the majority between 25 and 35 years. Most participants were female and had between one to 5 years of experience of providing maternity care.

### Emerging themes

The main emerging themes are presented below with illustrative quotes provided in Tables 1, 2 and 3.

**Table 1:** Enabling factors to screening for infective maternal morbidity

Sub-theme	Quote	
**Awareness of infectious morbidity**	Q1	“A pregnant woman is prone to infection” FGD P9 Midwife
	Q2	“Often we see infection after delivery” KII 12 Doctor
**Awareness of the severity of the effect of infections on the health of the mother**	Q3	“In the periphery (community) they tend to detect infections really late, so by the time they get here …. the patient is very sick” FGD P5 Doctor
	Q4	“So, by the time you realize they have infection the infection is already severe, and their life is at risk” KII 4 Nurse Midwife
	Q5	“Patients come back to hospital after they have recently been discharged home after delivery, some very sick, some even dead, and we believe most of them were discharged with an infection, but this was not recognised” KII 10 Doctor
**Current practice to detect infectious morbidity**	Q6	“We check if there are any vaginal sores, we also check for syphilis and treat any infection in the mother and the child plus the father” FGD P4 Nurse Midwife
	Q7	“When we triage, we check vital signs, in order to decide if the patient is an emergency patient, or a priority, or one that requires to be in the queue” KII10 Doctor
	Q8	“So normally in our department we do our own triage system, yes, that is to check which patients are in need of urgent care, yes we do that” KII 4 Nurse Midwife
	Q9	“We use the CHEWA early warning score in the high dependency unit here” FGD 2 Nurse
	Q10	“I have heard of the SOFA score for sepsis” KII 6 Doctor
**The benefits of using an early warning score**	Q11	“The early warning tool for sure is very important because the more we are able to detect infection early the more we help save the lives of the mums and even the babies” FGD P8 Midwife
	Q12	“I think that screening tools are important, as they help to detect the early signs of infection in a patient.” KII 2 Doctor
	Q13	“An early warning score will guide us to the management of the patient” FGD P8 Nurse Midwife
	Q14	“Earlier detection [of infection] equals to earlier treatment equals to earlier discharge and this prevents ‘congestions on the postnatal ward” KII 3 Doctor

^a^*KII* Key informant interview, *FGD* Focus group discussion

**Table 2:** Barriers to the provision of use of an early warning score

Sub-theme	Quote	
**Lack of resources**	Q14	“Sometimes people may neglect to check the vital signs as there aren’t the resources available” KII 7 Doctor
	Q15	“On labour ward our main challenge is lack of resources, we may find that for a whole day we don’t have a thermometer” FGD P7 Midwife
	Q16	“There may not be any thermometers there, sometimes you find there aren’t the tools to do what you want” KII 10 Doctor
	Q17	“Resources are a problem” FGD 8 Nurse Midwife
	Q18	“Sometimes there are no batteries for the electronic blood pressure machines” FGD 1 Nurse Midwife
	Q19	“There is lack of labs tests and the white cell count takes several hours to be done” FGD 2 Nurse
	Q20	“We need a paper supply for the observations charts” FGD 1 Nurse
**Lack of trained staff and time**	Q21	“The large number of patients with a small number of staff means that we are always short of time, and it may be difficult to assess each woman in full” KII7 Doctor
	Q22	“So maybe there are two nurses so really to monitor patients like they are supposed to its not really a simple task sometimes patients are missed” KII4 Nurse Midwife
	Q23	“Short staff time because you find there are many patients” FGD 8 Nurse Midwife
**Lack of understanding of how to interpret the score**	Q24	“You can have the tools or scores but if you don’t have knowledge on how to interpret these then there is nothing you have done” FGD 2 Midwife
	Q25	“Focusing on the score only can, in rare cases, result in overseeing other issues” KII 8 Doctor
**Lack of freely available medication**	Q26	“Infection may be detected but patients may not have funds to buy treatment” FGD 1 Nurse

^a^*KII* Key informant interview, *FGD* Focus group discussion

**Table 3:** Suggested solutions for the provision of the use of an early warning score

Sub-theme	Quote	
**Usefulness of colour codes**	Q27	“If it could be printed in colour, using a traffic light system, then one should be able to interpret findings very quickly” FGD P9 Nurse Midwife
**Education of healthcare providers**	Q28	“Orientation to the screening tool is needed so everyone understands it well “KII6 Nurse Midwife
	Q29	“So sometimes with the staff, you forget there are new people who can’t use it until they are trained” KII8 Doctor
	Q30	“I think with appropriate training we can use an early warning tool or score, it is such a simple tool to use” KII1 Doctor
**Recognition of the need to screen and using an early warning score**	Q31	“If each and every one would know the importance of screening that would help - some people may not know its importance now” KII7 Doctor
	Q32	“Yes, I think we (as a team) should be able to use an early warning score or tool. The fact that the midwives in the labour ward high dependency unit use it already means that everyone can actually use it” KII8 Doctor
	Q33	“With appropriate training it [an early warning score] could be used more widely in our department. it could be implemented everywhere in hospital” KII9 Doctor
	Q34	“It’s now about training everyone, orienting everyone, on how to effectively use it … because that tool is as good as the information you put on it” KII1, Doctor
**Local clinical leadership and use of audit**	Q35	“We need dedicated staff to pioneer it [early warning score] as happened in the high dependency unit, the introduction of score in other areas [of the hospital] have failed previously” KII 1 Doctor
	Q36	“If doctors haven’t welcomed the idea, they will stick to the process they learnt in college” FGD 7 Nurse
	Q37	“[We need to] use audits before and after to see what the issues are in implementation [of the early warning scores]” KII Doctor

^a^*KII* Key informant interview, *FGD* Focus group discussion

### Knowledge and attitude to screening for infectious morbidity during and after pregnancy

Healthcare providers were aware that women were at high risk of infective morbidity both during and after pregnancy (Table 1, Q1–2) and were aware that infective maternal morbidity includes a spectrum of disorders which vary in severity (Table 1, Q3–4). Healthcare providers had knowledge and experience of infectious morbidity in women not being recognized and leading to severe and life-threatening infections and sepsis especially where women had been discharged after birth and had to be readmitted in the postnatal period (Table 1, Q5). Healthcare providers reported that current approaches to screening for infection included: physical examination, checking vital signs as a form of triage, the ChEWA (the Chantinka Early Warning Alarm) screening tool in the high dependency unit and one participant mentioned knowledge of the SOFA (Sequential Organ Failure Assessment) score for detection of sepsis (Table 1, Q6–10). Many healthcare providers were aware of the benefits of using an early warning score as an approach to screening for infection (Table 1, Q11–14). There was an underlying willingness of the healthcare providers to provide better care. However, there were significant barriers in place.

### Barriers to implementation of screening for infectious morbidity during and after pregnancy

Reported challenges to the comprehensive assessment of infectious maternal morbidity included lack of simple equipment such as a thermometer, a lack of time with high patient volume and a small staff complement, staff not trained to use early warning tools or scores other clinical emergencies taking priority (Table 2, Q14–20). Many healthcare providers reported that a lack of time due to the large number of women attending for maternity care and the lack of simple equipment were challenges that made it difficult for them to screen and manage women who might have infectious morbidity but were not (yet) seriously sick (Table 2, Q21–23). Participants commented on the challenges to train and orientate staff in the interpretation of using early warning scores (Table 2, Q24–25); and that some patients may not be able to buy the treatment recommended if infection is detected (Table 2, Q26).

### Recommendations for change in practice

Healthcare providers were keen to discuss possible solutions and had recommendations regarding how to introduce screening as part of routine maternity care (including antenatal and postnatal care) with various approaches to implementation suggested. Healthcare providers were keen for the introduction of routine infection prevention health guidelines and a standardised questionnaire or triage system or an early warning score to help guide the assessment of women during antenatal and postnatal care, both in outpatient and inpatient settings. The use of visual aids such as posters and the use of colour to highlight severity (for example using a traffic light system: red, amber, green) were suggested as useful aids to implementation (Table 3 Q27). Many healthcare providers reported that training and orientation for new (and existing staff) would help (Table 3 Q28–30) with the implementation of an early warning score, and that screening for infection should be used in all departments of the hospital (Table 3 Q28–33).

Some participants reported that the adaptation of any triage system or early warning score to their own local setting was important, especially if the scoring system was originally designed and validated in high resource settings, as the resources in place in their setting (for example laboratory support) were much more limited. Participants emphasised that any additional screening approach would need to be perceived as a useful additional aide to help them care for their patients, instead of an additional cumbersome and ineffective ‘paper task’ to complete. They also felt a multidisciplinary team approach, collaborating with laboratory technicians and microbiologists as part of routine maternity care was important and encouraged a more comprehensive and holistic approach to maternity care. Local clinical leadership would benefit implementation and the use of clinical audit would demonstrate the effectiveness of such implementation to other areas of the hospital, including antenatal and postnatal departments (Table 3, Q34–36).

## Discussion

### Statement of principal findings

Healthcare providers are aware of the problem and impact of maternal morbidity and mortality due to different types of infection during pregnancy and after childbirth. Healthcare providers understand that maternal morbidity related to infection should be specifically assessed and can present at any time from early pregnancy through to the late postnatal period. Early identification of infectious morbidity can be difficult if healthcare providers can only rely on the overall clinical condition of women and/or on women’s self-reporting of symptoms. However, ‘routine’ screening of women for infectious maternal morbidity during and after pregnancy as part of routine maternity care is not in place due to a lack of training, lack of time, and lack of free and easily available diagnostic tools (including thermometers). Screening using an early warning score that was easy to use, colour coded, adapted to the local settings, and properly implemented was welcomed. Healthcare providers also reported needing support from the wider multidisciplinary team including laboratory technician as well as a re-focus on prevention and early identification of underlying maternal morbidity in addition to life-threatening conditions.

### Strengths and limitations of the study

Our study adds to existing evidence exploring the knowledge and attitude of healthcare providers regarding the potential usefulness and effectiveness of approaches to early detection of infectious maternal morbidity as part of routine maternity care in low resource settings where the burden of disease is high and unrecognized infection is known to be one of the causes of maternal and neonatal mortality. This study highlights barriers as well as solutions to inform programmes that seek to introduce and establish routine screening for infectious maternal morbidity during and after pregnancy in low resource settings. Practical recommendations were provided by a range of different cadres of healthcare providers (both female and male), who worked in different departments within a busy maternity setting and had varied level of experiences. All healthcare providers interviewed recognised this as an important area and were keen to embrace change in practice that would help reduce maternal and neonatal morbidity and mortality. This study population comprised mainly doctors and nurse-midwives providing routine maternity care in one large teaching hospital and the findings cannot be assumed to be the same in other hospitals and other settings. However, it may be assumed that resource limitations for community-based healthcare providers in different settings are similar or worse. This study used a qualitative study design and limitations are that findings cannot be generalised because of smaller sample sizes and context specificity; data analysis can be influenced by researchers’ personal perceptions and unconscious biases; and rigor is more difficult to maintain, assess, and demonstrate [38]. However, for this study we chose to use qualitative research methodology to understand the local context and influences; to listen to healthcare provider’s voices, experiences, and opinions; to explore in-depth knowledge, attitudes; and to understand perceptions of a proposed clinical intervention.

### How does this study relate to other literature?

In our study, many healthcare providers had knowledge of and were aware of the benefits of early warning scores, like other studies [39–41]. Healthcare providers were familiar with the use of an early warning score in the maternity high dependency unit as part of critical care, the ChEWA score which uses standard clinical observations such as pulse rate, respiratory rate, temperature, blood pressure, in combination with the mental state score (AVPU – alert, responds to visual stimuli, responds to painful stimuli, unresponsive) to detect the deteriorating unwell mother after childbirth [23]. The benefits of the use of early warning scores in our study was like a study in Ethiopia where healthcare providers were positive about the implementation of an early warning score, reported that this was easy to use and improved communication between nurses and doctors [40]. However, like our study and other studies conducted in low resource settings, healthcare providers reported challenges to implementation including regarding lack of staff, lack of time, lack of resources and lack of training on how to interpret early warning scores [41–44]. One study conducted in Malawi highlighted that healthcare providers reported that the time required to measure vital signs required to obtain an early warning score is too much and that healthcare providers would prefer to continue with a quick subjective clinical judgement only to identify if a patient has an infection or not [41]. Similar to findings in our study, local ownership and clinical leadership, good orientation, staff training and integration of clinical change into respective care bundles and local management guidelines are important for successful implementation [23, 40–45]. In our study, many healthcare providers did appreciate the benefits of screening for infection in women as part of routine antenatal and postnatal care, not just during labour and childbirth as part of critical care. However, similar to another study conducted in Uganda, in our study healthcare providers did emphasis the importance of ensuring any proposed change in clinical practice was adapted to the local setting and resources made available for any change to be adopted and effective, specifically highlighting the difficulty in using a blood result as a parameter for early warning score due to lack of rapid results from the laboratory [46]. There are portable rapid diagnostic testing devices for white cell count, lactate and C-Reactive protein available but assessment of their feasibility and cost effectiveness as screening for infection in routine maternity care in low resource settings is awaited [47–49]. The importance of the provision of free targeted treatment and further management if infection is detectedmust be available alongside the implementation of routine screening for infectious maternal morbidity. Any proposed single implementation must be contextually appropriate and complement current clinical care to support the provision of high quality comprehensive holistic maternity care [50].

### Unanswered questions

There are currently several international clinical and policy guidelines on who should enquire, screen, and manage infectious maternal morbidity during and after pregnancy as part of routine antenatal and postnatal care, and how this should be conducted, including identification, counselling, documentation, first line medication and provision of higher referral pathways [51, 52]. However, the practicalities associated with the introduction, implementation, sustainability of change and acceptability of these guidelines as part of routine maternity care in countries such as Malawi are currently uncertain. There is a need to better understand how infectious maternal morbidity can be prevented, detected using early warning scores, investigated, and treated across different healthcare levels in low resource settings. There is also debate as to who is most suitable to enquire, screen for and manage infectious maternal morbidity and at what level of the health system (community, primary or secondary health care level) across LMIC. In many high-income countries, specially trained nurses or midwives routinely assess, support, and provide further referral between different levels of care. This approach in a low resource setting requires further research and evaluation.

## Conclusion

Routine screening and early detection of infectious morbidity during and after pregnancy should be considered a key component of routine maternity care to reduce the burden of maternal and neonatal morbidity and mortality resulting from infections. Many healthcare providers are keen to be given the tools to detect infection early and for resources to be made available to them to do so. This study highlights the need to understand the complexity of factors associated with a change in clinical practice and provides practical recommendations to developing better screening approaches as part of routine maternity care in low resource settings.

## Supplementary Information

**Additional file 1.**

## Data Availability

The dataset used and analysed during the current study are available from the corresponding author on reasonable request.

## Abbreviations

ANC: Antenatal care
AVPU: Alert, Visual, Pain Unresponsive
ChEWA: Chantinka Early Warning Alarm
KII: Key Informant Interview
FGD: Focus Group Discussion
LMIC: Low- and Middle-Income Countries
PNC: Postnatal Care
SOFA: Sequential Organ Failure Assessment
UK: United Kingdom
WHO: World Health Organization
