# Supplementary material for: Screening for infectious maternal morbidity - knowledge, attitudes and perceptions among healthcare providers and managers in Malawi: a qualitative study
Source: BMC Pregnancy Childbirth. 2022 Apr 26;22:362. doi: 10.1186/s12884-022-04583-5 (PMC9040689; doi:10.1186/s12884-022-04583-5)
Supplement: Supplementary file 1 — Additional file 1. [file 12884_2022_4583_MOESM1_ESM.pdf]

SCREENING FOR INFECTIOUS MATERNAL MORBIDITY - KNOWLEDGE ATTITUDES AND PERCEPTIONS AMONG HEALTHCARE PROVIDERS AND MANAGERS IN MALAWI: A QUALITATIVE STUDY

Authors:

SLEZAK, Emilia[1]

UNGER, Holger [1, 2]

GADAMA, Luis[3]

MCCAULEY, Mary[1,4]*

[1]Centre for Maternal and Newborn Health, Liverpool School of Tropical Medicine, United Kingdom

[2]Department of Obstetrics and Gynaecology, Royal Darwin Hospital and the Menzies School of Health Research, Darwin, Australia

[3]Queen Elizabeth Hospital, Blantyre, Malawi

[4]Liverpool Women's Hospital, Liverpool, United Kingdom

*Corresponding author:

mary.mccauley1@lwh.nhs.uk

Liverpool Women's Hospital

Crown Street, Liverpool

L8 7SS

Topic guide for Focus Group Discussion

(Also translated in Chichewa)

FOCUS GROUP: DEMOGRAPHIC DETAILS QUESTIONNAIRE

Please answer the following questions in the spaces provided, circle or tick the most appropriate options.

1. What is your professional?

- □ Midwife or Nurse Midwife
- □ Nurse
- □ General Doctor
- □ Medical officer
- □ Other: (please describe) ___________________________________

What is the name of your place of work?

____

Thank you for taking the time to complete this questionnaire

FOCUS GROUP: DISCUSSION GUIDE

Facilitator's welcome, introduction and instructions to participants

Welcome and thank you for volunteering to take part in this focus group. You have been asked to participate as your point of view is important for this research that is part of a much larger project. I realize you are busy and I appreciate the time you have given.

Introduction: This focus group discussion is designed to assess your current thoughts and feelings about the use of an early warning screening tool to detect infection in women during and after pregnancy. The focus group discussion will take no more than 1 hour. May I tape the discussion to facilitate its recollection? (if yes, switch on the recorder)

Anonymity: Thank you for signing the consent form. Although this discussion is being taped, I would like to emphasize that this is anonymous and that this is being recorded on a locked devise that only myself knows the password to. And once transcribes have been made, the audio recordings will be deleted. The transcribes will not contain any information that identify you. Please try and be as honest as you can and try and refrain from discussing anything said in this group outside of this group and if there are any questions that you don't want to answer you do not have to. If you want to leave the study at any time you can and if you want to leave the room you can.

Ground rules

- Only one person speaks at a time. There may be a temptation to jump in when someone is talking but please wait until they have finished.
- There are no right or wrong answers
- You do not have to speak in any particular order
- When you do have something to say, please do so. It is important that I obtain the views of each of you
- You do not have to agree with the views of other people in the group
- For the purpose of this study, if you can please speak in English. If you feel comfortable speaking in Chichewa, feel free. I have an interpreter here who will help to facilitate and translate this discussion.
- Does anyone have any questions? (answers).
- OK, let's begin

Warm up

- First, I'd like everyone to introduce themselves.

Introductory question

I am very interested to hear about your general views regarding what approaches to screening for infection you know for women that you care for during and after pregnancy.

In addition, I am keen to understand how best to screening women for infection during and after pregnancy in your setting.

I would like to understand your perceptions regarding the possible barriers or enabling factors to routinely screen women for infection during and after pregnancy in your setting.

I am just going to give you a couple of minutes to think about_about, How the use of an early warning screening tool to detect infection in women during and after pregnancy could be used in your health care facility/ in a primary health care facility. If you want to leave the study at any time you can and if you want to leave the room you can.

How the use of an early warning screening tool to detect infection in women during and after pregnancy could be used in your health care facility/ in a primary health care facility.

Guiding questions

- Have you used any screening tools or triage systems in your clinical practice?
- What are the thoughts of you and other staff towards the use of the early warning screening tool? (What did people think/say/do?)
- How do you screen for infection and how do you diagnose infection in your setting?
- What do you think about the early warning screening tools I have given you to look at?
- Do you think that the use of an early warning screening tool is likely to improve the safety and patient outcomes and efficiency of women with maternal infections?
- Do you think that you would be able to use it as a team in your work place specific to health care workers?
- What do you think are the main issues around using an early warning screening tool?
- What barriers do you think there are in using an early warning screening tool in your setting/ in your facilities that you represent?
- What drove the positive/negative reaction? If negative, how could it be rectified?
- Would you feel comfortable using it?
- How easy would it to adapt it?
- What was your knowledge before about using an early warning screening tool?

Concluding question

- Of all the things, we've discussed today, what would you say are the most important issues you would like to express about an early warning score?
- How would you explain the concept of the screening tool?

Conclusion

- Thank you for participating. This has been a very successful discussion
- Your opinions will be a valuable asset to the study
- We hope you have found the discussion interesting
- If there is anything you are unhappy with or wish to complain about, please contact the local principal investigator (Dr Luis Gadama) or speak to me later there are further details on your information sheets.
- I would like to remind you that any comments featuring in this report will be anonymous
- Before you leave, please hand in your completed personal details questionnaire

Topic guide for Key Informant Interviews

(Also translated into Chichewa)

INTERVIEW QUESTIONAIRE.

Please answer the following questions in the spaces provided, circle or tick the most appropriate options.

1. What is your professional?

- □ Midwife or Nurse Midwife
- □ Nurse
- □ General Doctor
- □ Medical officer
- □ Other: (please describe) ___________________________________

What is the name of your place of work?

____

Thank you for taking the time to complete this questionnaire

Key Informant Interview: DISCUSSION GUIDE

Facilitator's welcome, introduction and instructions to participants

Welcome and thank you for volunteering to take part in this Interview. You have been asked to participate as your point of view is important for this research that is part of a much larger project. I realize you are busy and I appreciate the time you have given.

Introduction: This Interview is designed to assess your current thoughts and feelings about the use of an Early warning screening tool to detect infection in women during and after pregnancy. This interview will take no more than 30-45 mins. May I tape the discussion to facilitate its recollection?

(if yes, switch on the recorder)

For the purpose of this study, if you can please speak in English. If you feel comfortable speaking in Chichewa, feel free. I have an interpreter here who will help to facilitate and translate this discussion.

Anonymity: Thank you for signing the consent form. Although this discussion is being tapped, I would like to emphasise that this is anonymous and that this is being recorded on a locked devise that only myself knows the password to. And once transcribes have been made will be deleted. The transcribes will not contain any information that identify you. Please try and be as honest as you can and try and refrain from discussing anything said in this group outside of this group and if there are any questions that you don't want to answer you don't have to.

Introduction

I am very interested to hear about your general views regarding what approaches to screening for infection you know for women that you care for during and after pregnancy.

In addition, I am keen to understand how best to screening women for infection during and after pregnancy in your setting.

I would like to understand your perceptions regarding the possible barriers or enabling factors to routinely screen women for infection during and after pregnancy in your setting.

I am just going to give you a couple of minutes to think about, **<u>How the use of an early warning screening tool to detect infection in women during and after pregnancy could be used in your health care facility/ in a primary health care facility.</u>** If you want to leave the study at any time you can and if you want to leave the room you can

Guiding questions

- Have you used any screening tools or triage systems in your clinical practice?
- What are the attitudes of you and other staff towards the use of the early warning screening tool? (What did people think/say/do?
- How do you screen for infection and diagnose infection in your setting?
- What drove the positive/negative reaction? If negative, how could it be rectified?
- What do you think about the early warning screening tools I have given you to look at?

- Do you think that the use of an early warning screening tool is likely to improve the safety and patient outcomes and efficiency of women with maternal infections?
- Do you think that you would be able to use it as a team in your work place specific to health care workers)?
- What do you think are the main issues around using an early warning screening tool?
- What barriers do you think there are in using an early warning screening tool in your setting/ in your facilities that you represent?
- Would you feel comfortable using it?
- How easy would it to adapt it?
- What was your knowledge before about using an early warning screening tool?

Concluding question

- Of all the things we've discussed today, what would you say are the most important issues you would like to express about an early warning score?
- How would you explain the concept of the screening tool?

Conclusion

- Thank you for participating. This has been a very successful interview
- Your opinions will be a valuable asset to the study
- I hope you have found the interview interesting
- If there is anything you are unhappy with or wish to complain about, please contact the local principle investigator (Dr Luis Gadama) or speak to me later there are further details on your information sheets.
- I would like to remind you that any comments featuring in this report will be anonymous
- Before you leave, please hand in your completed personal details questionnaire
